# Does geopolitical risks drive the extreme spillovers of bulk energy and chemical commodity

**DOI:** 10.1371/journal.pone.0326268

**Published:** 2025-06-18

**Authors:** Zhang Tao, Tianzhuang Li, Yadi Chen, Xiaoyue Huang

**Affiliations:** 1 Guangxi Colleges and Universities Key laboratory of Intelligent Logistics Technology, Nanning Normal University, Nanning, Guangxi, China; 2 School of Management and Economics, Nanning Normal University, Nanning, Guangxi, China; 3 The Institute for Sustainable Development, Macau University of Science and Technology, Macao, China; University of Tunis El Manar Faculty of Economic Sciences and Management of Tunis: Universite de Tunis El Manar Faculte des Sciences Economiques et de Gestion de Tunis, TUNISIA

## Abstract

Geopolitical risks (GPR) can affect the prices of natural resources, which are crucial for survival and sustainable economies. Based on the TVP-VAR-BK models, this paper examines the asymmetric risk contagion between geopolitical risk, bulk energy, and chemical commodity from a time-frequency perspective. The results show that the risk contagion of geopolitical risks, bulk energy and chemical commodity markets has time-varying asymmetry and periodicity, and is susceptible to extreme events. Furthermore, the main driving factor is high-frequency spillover, which has “short-term fragility”, that is, the risk spillover in the short term is more significant than in the medium and long term, and network spillover is mainly caused by negative risk changes. In addition, the bulk energy market is the main risk transmitter, while the chemical commodity market is the main risk receiver. The risk contagion of crude oil on bitumen markets is the most significant. Finally, as a downstream market of the crude oil industry, the chemical commodity market not only directly accepts risk contagion from the crude oil market, but also transmits risks along the “bulk energy - geopolitical risk - chemical commodity” path. This evidence has important implications for governments and regulators in taking steps to prevent extreme spillover effects and a series of chain reactions from geopolitical risks.

## 1. Introduction

Geopolitical risk (GPR) has emerged as a major source of exogenous uncertainty in global commodity markets, exerting profound influence on price dynamics, investment behavior, and supply chain resilience [1]. With the continuous integration of bulk energy and financial markets, the financialization level of the energy market has been deepening [2]. Consequently, geopolitical shocks, such as regional conflicts, trade disputes, and sanctions, often trigger price co-movements in the energy and chemical markets, leading to cascading volatility and elevated systemic risk. The outbreak of the Russia-Ukraine conflict in 2022 soared geopolitical risks, and prices for bulk energy and chemical commodities began to spiral upward [3]. As the world’s largest crude oil importer, China faces heightened exposure to external shocks transmitted through global commodity markets. These shocks not only increase industrial production costs, but also amplify financial volatility, potentially destabilizing the macroeconomy [4]. Therefore, based on the perspective of the petrochemical industry chain, this paper analyzes the tail risk contagion between geopolitical risks, bulk energy, and chemical commodity markets, which provides reference for cross-market risk prevention.

Geopolitical uncertainty is one of the important driving factors for the continuous volatility and shocks of bulk energy and chemical commodities [2], which have broad implications for trading decisions [5] and investor sentiment in commodity markets [6]. Therefore, the long-term stability of bulk energy and chemical commodity markets will be impacted by the panic of investors in response to uncertainty and extreme events caused by geopolitical risks [7]. The current academic and policy authorities have shifted their attention to the issue of maintaining the stability of Chinese bulk energy and chemical commodity markets in response to the impact of geopolitical risks.

Recent research has devoted increasing attention to risk transmission across interconnected markets. Based on methodologies such as VAR models [8,9], GARCH models [10], DY spillover indices [11], and Copula function [12], many studies have confirmed the link between commodity price fluctuations and geopolitical uncertainty [13]. Nonetheless, given that market volatility is influenced by both persistent macroeconomic and transient uncertainty shocks [14,15], the characteristics of cross-market spillovers vary across temporal horizons and market phases. Therefore, in order to cope with the impact of geopolitical risks and propose measures to maintain the stability of bulk energy and chemical commodity markets in China, this study employs TVP-VAR-BK models to explore the tail risk dynamics among geopolitical risk, bulk energy, and chemical commodities at multiple time scales and under both bullish and bearish conditions. Furthermore, a complex network framework is introduced to comprehensively characterize the systemic transmission pattern and provide practical suggestions for mitigating cascading risks in the commodity system. The results reveal that the risk spillover between geopolitical risks, bulk energy and chemical commodity markets presents a risk transmission structure of “upstream active to downstream passive”. Additionally, there is significant time variation and frequency-domain heterogeneity, and the high-frequency spillover effect dominates the risk transmission. At the same time, the risk spillover effect is asymmetric, with negative shocks being stronger than positive shocks. Furthermore, under negative economic expectations, linear low-density polyethylene (LLDPE) transforms from a risk receiver to a risk transmitter, and is highly sensitive to changes in macroeconomic expectations.

The marginal contributions are as follows: First, most existing studies have discussed the volatility contagion between geopolitical risks and isolated commodities [7,16]. This paper explores the risk contagion between the geopolitical risks, bulk energy, and chemical commodity markets from the perspective of the petrochemical industry chain, which helps market participants systematically understand the risk transmission mechanisms and network structures. Second, since risk contagion has time-varying asymmetry and periodicity evolution [17,18] under different time scales and market conditions [19], this paper examines the risk contagion characteristics of geopolitical risks, bulk energy and chemical commodity markets in both tranquil and turbulent periods, which helps market participants comprehensively grasp the dynamic changes in network spillover paths and risk contagion sources. Finally, this paper uses TVP-VAR-BK models to divide the data into positive and negative volatility, characterize the time-varying asymmetry and periodic evolution of spillover effects, and analyze network spillover paths and risk contagion sources by using complex network methodology. This accurately identifies the cross-market risk transmission path and reveals the heterogeneity of volatility spillover under different market conditions.

## 2. Literature review

### 2.1. Geopolitical risk research

GPR (geopolitical risk) is the term used to describe the occurrence and escalation of conflicts, terrorism, and any other tense situation that disrupts international peaceful relations between states or political entities [20]. Since the new century, the probability of GPR entering the high fluctuation interval has been increasing [21], and its impact on global economic dynamics has emerged as a critical concern [22]. As a form of macroeconomic and political uncertainty, GPR is considered to be the main determinant of production behavior, industrial progress and business cycle, which strongly correlate to the performance of bulk energy and chemical commodities [23,24]. Early scholars focused on employing event studies to qualitatively analyze the linkage between GPR and bulk energy and chemical commodities. Zhang et al. [25] studied the impact of major emergencies, namely the Iraq War, the Iranian Revolution and the Gulf War on oil prices. Ramiah et al. [26] employed an improved event study method to explore the impact of recent terrorist attacks on commodity markets. They found that these attacks increase the uncertainty within the commodity market. However, due to the limitations of sample size and data quality, the conclusions drawn from these studies lack convincing evidence. The geopolitical risk (GPR) index proposed by Caldara and Iacoviello in 2018 [27] just makes up for this deficiency, triggering quantitative research upsurge on GPR in academia. Chowdhury et al. [28] used quantile on quantile regression (QQR) to concluded that GPR exerts a significant negative impact on global grain and energy markets. Zheng et al. [10] adopted GARCH model to analyze the impact of GPR on commodity markets. The results show that GPR significantly increases futures price volatility on coal, iron ore and crude oil, and significantly reduces the gold prices volatility; However, it has no remarkable effect on the fluctuation of copper futures price.

### 2.2. The volatility contagion of bulk energy and chemical commodity markets

The bulk energy and chemical commodity markets have obtained recent academic attention owing to their considerable price fluctuations [29–31]. The initial concern over commodity price volatility comes from the seminal research of Pindyck and Rotemberg [32], who held that commodity prices exhibit similar trends, and therefore may have a volatility linkage. This finding provides a new research direction, scholars start to investigate the spillover effects between commodity prices, returns and volatility. Baffes [33] studied the transmission degree of crude oil price fluctuations to other 35 primary commodities prices. He suggested that the fertilizer index had the highest pass-through in the non-energy index, followed by the food commodity index, while precious metals also exhibited a strong response to crude oil price. This indicates that sustained high crude oil prices will likely lead to a substantial and prolonged rise in commodity prices. Wang et al. [34] measured the return spillovers between the Chinese and American stock markets and the international commodity markets, and discussed the dominant factors influencing these cross-market spillovers combined with actual demand and speculation. It showed that the impact of speculation on return spillover was much greater than that of actual demand. Based on the volatility spillover index under the TVP-VAR framework, Wen et al. [35] examined the dynamic volatility spillover between Chinese stock market and commodity market, highlighting the significant spillover effects of nonferrous metals and chemical industries on the stock market. In addition, Wang et al. [3] also found that the level of GPR in high returns and volatility spillovers is relatively high. In the crisscross industrial chain of bulk energy and chemical commodities, price fluctuations will affect not only the commodity in question, but also cause a chain effect between other commodities [36]. Consequently, the volatility spillover effect and information transmission mechanism within bulk energy and chemical commodity markets need to be further explored [2].

### 2.3. The volatility contagion of geopolitical risk and bulk commodity markets

GPR and bulk commodity markets are always hot topics in their respective fields and have been a longstanding concern. Studies on the crossing of the two have begun early, but there are fewer scholars who were originally concerned about it. Li et al. [37] used the spillover index model to explore the dynamic spillover effect between GPR and gold prices in 18 emerging countries. The results show a positive spillover effect between GPR and gold prices, with the net spillover originating from the impact of GPR on gold price. Polat et al. [38] investigated the dynamic linkage between GPR and the agricultural product markets, and their TVP-VAR structure indicated that both wheat and GPR would transmit volatility shocks. Gong and Xu [2] regarded commodities as a whole, revealing the dynamic relationship between GPR and the five commodity markets, namely energy, precious metals, industrial metals, agriculture and animal husbandry. They found that GPR would significantly affect the overall correlation of commodity markets, and GPR had different effects on the net spillover of different commodity markets. However, it should be noted that the risk spillover between GPR and bulk energy and chemical commodity markets will show different characteristics in different frequency domains. Jin et al. [39] comprehensively analyzed the static and dynamic spillovers between GPR and energy market across different frequency domains, and pointed out that the static spillover was more obvious at low frequencies, on the contrary, the dynamic spillover was more obvious at high frequencies. Gong et al. [16] obtained similar conclusions by analyzing the cycle characteristics of the international crude oil market and the GPR dynamic network. They found that the short-term risk contagion effect was more obvious. However, some scholars hold the opposite view. Coskun et al. [11] examined the volatility correlation between geopolitical oil price risk, clean energy stocks, global stocks and commodity markets. It showed that long-term volatility correlation plays the most dominant role in the system, indicating that the volatility spillover effect among markets is persistent and long-lasting.

Moreover, the spillover effect between GPR and bulk commodity markets is also asymmetric and heterogeneous, and many previous studies have provided conclusive evidence. In terms of asymmetry, Qin et al. [40] adopted the quantile regression model to investigate the asymmetric impact of GPR on the energy market return and volatility under different market conditions. They found that GPR had a significant negative impact on crude oil returns during the bear market and on heating oil returns in the bull market, but had no effect on natural gas returns. In the volatility transmission, the impact of GPR on crude oil volatility is positive in different market conditions, while the impact on natural gas and heating oil is mainly negative. Cheng et al. [18] studied the effects of GPR on the gold-oil relationship, and believed that the presence of nonlinear and asymmetric effects between GPR and gold-oil relationship. In terms of heterogeneity, Li et al. [41] argued that the impact of GPR on Chinese grain prices is prolonged and vary according to different grain varieties. Specifically, the shock of GPR to soybean prices is the greatest, followed by corn and wheat prices, and the last is japonica rice prices. Other scholars examined the commodity market as a whole, and revealed that prices of different commodities have a certain degree of heterogeneity in the response to GPR [15]. Specifically, GPR has a positive impact on the net spillover of energy, agriculture and animal husbandry commodity markets, and a negative impact on precious metals and industrial metal commodity markets [2].

In summary, at the theoretical level, the existing literature has not yet developed a systematic theoretical logic that integrates the transmission path of the entire industry chain. Additionally, the asymmetric fluctuation characteristics of GPR lack interdisciplinary theoretical support. At the empirical level, existing research mainly focuses on the static relationship or bivariate spillover effect between GPR and specific commodities, but lacks the dynamic evolution characteristics and network structure analysis of risk transmission. Therefore, based on the perspective of the petrochemical industry chain, this paper uses the TVP-VAR-BK models, as well as complex network analysis, to study the contagion mechanism and network structure of risks between GPR, bulk energy, and chemical commodity markets under different time scales (short, medium, and long term) and market conditions (market rise and fall). This not only theoretically constructs a fusion analysis framework of “Industry chain - Time-frequency spillover - Market risk sensitivity”, but also empirically quantifies the risk transmission path, core contagion source and dynamic evolution characteristics from the perspective of the industrial chain. Based on the above viewpoints, this paper proposes the following related hypotheses:

H1: The volatility spillover between geopolitical risks and commodity markets has dynamic characteristics.

H2: Market risk spillover networks are asymmetric under shocks.

H3: Geopolitical risk, bulk energy, and chemical commodity markets exhibit structural mutations.

## 3. Methodology and data

### 3.1. Methodology

#### 3.1.1. TVP-VAR-DY.

When analyzing time series data, it is essential to consider their dynamic characteristics, particularly the temporal evolution of indicators. The rolling window model is one of the most commonly used and widely applied analytical methods. However, conventional rolling window settings may lead to sample loss and induce instability in model results. The DY spillover index model [42] shows highly sensitive to outliers. To address these issues, Antonakakis et al. [43] integrated the TVP-VAR-SV model with the DY spillover index model and proposed the TVP-VAR-DY model.

The development of the TVP-VAR-DY model can be divided into several key steps: first, the TVP-VAR(p) model can be expressed as:


$$\begin{array}{c} \begin{array}{c} y_{t}=B_{t}\beta_{t-1}+\varepsilon_{t},\;\varepsilon_{t}|\Omega_{t-1}\sim N\left(0,\Sigma_{t}\right) \end{array} \end{array}$$ (1)

Here, $y_{t}$ is an N × 1 dimensional vector, and $B_{t}$ and $\Sigma_{t}$ denote the parameter matrix and the time-varying variance-covariance matrix, respectively. The TVP-VAR(p) model is then estimated using the Kalman filter algorithm, followed by an H-step-ahead forecast based on the estimated model. Subsequently, the Generalized Forecast Error Variance Decomposition (GFEVD) is conducted, as given by the following formula:


$$\begin{array}{c} \begin{array}{c} y_{t}=B_{t}\beta_{t-1}+\varepsilon_{t}=\sum_{i=0}^{\infty }A_{ij}\varepsilon_{t-i} \end{array} \end{array}$$ (2)

$A_{ij}$ denotes the impulse response function. Based on the Generalized Forecast Error Variance Decomposition (GFEVD), the spillover effect from variable I to variable j is defined as follows:


$$\begin{array}{c} \begin{array}{c} \varphi_{ij,t}(H)=\frac{\sum_{jj,t}^{-1}\sum_{h=0}^{H-1}{\left(\theta_{i}^{\prime }A_{h,t}\Sigma_{t}\theta_{j}\right)}^{2}}{\sum_{h=0}^{H-1}\left(\theta_{j}^{\prime }A_{h,t}\Sigma_{t}A_{h,t}^{\prime }\theta_{i}\right)} \end{array} \end{array}$$ (3)

$\Sigma_{jj,t}$ denotes the time-varying standard deviation of the j-th, and $\theta_{i}$ is the selection vector. To ensure the comparability of the variance decomposition matrix, normalization is required:


$$\begin{array}{c} \begin{array}{c} {\tilde{\varphi }}_{ij,t}(H)=\frac{\varphi_{ij,t}(H)}{\sum_{i=1,j=1}^{N}\varphi_{ij,t}(H)} \end{array} \end{array}$$ (4)

The total spillover index is obtained by summing all off-diagonal elements of the normalized variance decomposition matrix and dividing by the total number of variables, thereby quantifying the overall degree of risk transmission within the system.


$$\begin{array}{c} \begin{array}{c} {TCI}_{t}(H)=\frac{\sum_{j=1,i\neq j}^{N}{\tilde{\varphi }}_{ij,t}(H)}{\sum_{i=1,j=1}^{N}{\tilde{\varphi }}_{ij,t}(H)}\times 100 \end{array} \end{array}$$ (5)

To evaluate the extent to which a single variable acts as a source of risk to other variables in the system, as well as the degree to which it receives risk from other markets, this study incorporates the TO and FROM indicators. The TO indicator measures the extent to which a given variable influences other markets. A high TO value for a market indicates strong risk spillover effects on other markets, reflecting its potential to transmit systemic risk. The FROM indicator, on the other hand, captures the extent to which a variable is affected by other markets. A high FROM value suggests that the market is more susceptible to external risk transmissions.


$$\begin{array}{c} \begin{array}{c} {TO}_{i\to *,t}(H)=\frac{\sum_{j=1,j\neq i}^{N}{\tilde{\varphi }}_{ji,t}(H)}{\sum_{i=1,j=1}^{N}{\tilde{\varphi }}_{ij,t}(H)}\times 100 \end{array} \end{array}$$ (6)

$$\begin{array}{c} \begin{array}{c} {FROM}_{i\leftarrow *,t}(H)=\frac{\sum_{j=1,i\neq j}^{N}{\tilde{\varphi }}_{ij,t}(H)}{\sum_{i=1,j=1}^{N}{\tilde{\varphi }}_{ij,t}(H)}\times 100 \end{array} \end{array}$$ (7)

In addition, the NET indicator represents the net spillover effect, defined as the difference between the spillover that a variable transmits to others and the spillover it receives from others. This measure helps identify the net direction of risk transmission for a given variable within the system. When the TO value exceeds the FROM value, the variable is considered a net risk transmitter within the system. Conversely, when the FROM value exceeds the TO value, the variable acts as a net risk receiver.


$$\begin{array}{c} \begin{array}{c} {NET}_{i,t}(H)={TO}_{i\to *,t}(H)-{FROM}_{j\leftarrow *,t}(H) \end{array} \end{array}$$ (8)

Building on the analysis of net spillover effects between variables, the Net Pairwise Directional Spillover Index can be calculated as follows:


$$\begin{array}{c} \begin{array}{c} {NPDC}_{ij,t}(H)=\left(\frac{{\tilde{\varphi }}_{ji,t}(H)}{\sum_{i=1,j=1}^{N}{\tilde{\varphi }}_{ij,t}(H)}-\frac{{\tilde{\varphi }}_{ij,t}(H)}{\sum_{i=1,j=1}^{N}{\tilde{\varphi }}_{ij,t}(H)}\right)\times 100 \end{array} \end{array}$$ (9)

#### 3.1.2. TVP-VAR-BK.

Markets respond to economic shocks differently across frequencies, making it essential to investigate spillover effects between variables at different frequencies. Building on the seminal work of Diebold and Yilmaz [44], Barunik and Krehlik [45] developed spillover indices across different frequencies. Subsequently, Barunik and Ellington [46] integrated the BK spillover methodology with the TVP-VAR model to propose the TVP-VAR-BK model, which reveals spillover effects across different frequencies and further enhances our understanding of economic interdependencies. The construction of the TVP-VAR-BK model involves the following steps:

The frequency response function is defined as:


$$\begin{array}{c} \begin{array}{c} A_{t}\left(e^{-i\omega h}\right)=\sum_{h=0}^{H-1}e^{-i\omega h}A_{h,t} \end{array} \end{array}$$ (10)

Let i =$\sqrt{-1}$, the general causal spectrum across frequencies ω in the range of (− π, π) is formulated as:


$$\begin{array}{c} \begin{array}{c} {\left(f_{t}(\omega )\right)}_{ij}=\frac{\sum_{jj,t}^{-1}{\left|\theta_{i}^{\prime }A_{t}\left(e^{-i\omega }\right)\Sigma_{t}\theta_{j}\right|}^{2}}{\theta_{i}^{\prime }A_{t}\left(e^{-i\omega }\right)\theta_{j}} \end{array} \end{array}$$ (11)

Equation (11) describes how a shock originating from variable i affects the spillover to variable j at frequency ω. The frequency band is defined as (a, b), where a < b and a, b∈(-π,π). At frequency band d, the Generalized Forecast Error Variance Decomposition (GFEVD) is given by:


$$\varphi_{ij,t}(d)=\frac{1}{2\pi }\int_{a}^{b}W_{i,t}(\omega ){\left(f_{t}(\omega )\right)}_{ij}d\omega$$

Here, $W_{i,t}(\omega )=\frac{\theta_{i}^{\prime }A_{h}(e^{-i\omega }Sigma_{t}A_{h}^{\prime }(e^{-i\omega })\theta_{i}}{\frac{\pi }{2}\int_{-\pi }^{\pi }\left(\theta_{i}^{\prime }A_{h}(e^{-i\omega })\Sigma_{t}A_{h}^{\prime }(e^{-i\omega })\theta_{i}\right)d\omega }$. Similarly, to ensure comparability across frequency bands, normalization is performed as follows:


$$\begin{array}{c} \begin{array}{c} {\tilde{\varphi }}_{ij,t}(d)=\frac{\varphi_{ij,t}(d)}{\sum_{i=1,j=1}^{N}\varphi_{ij,t}(\infty )} \end{array} \end{array}$$ (12)

Therefore,


$$\begin{array}{c} \begin{array}{c} {TCI}_{t}(d)=\left(\frac{\sum_{i=1,j=1}^{n}{\tilde{\varphi }}_{ij,t}(d)}{\sum_{i=1,j=1}^{n}{\tilde{\varphi }}_{ij,t}(\infty )}-\frac{Tr\left\{{\tilde{\varphi }}_{ij,t}(d)\right\}}{\sum_{i=1,j=1}^{n}{\tilde{\varphi }}_{ij,t}(\infty )}\right)\times 100 \end{array} \end{array}$$ (13)

$$\begin{array}{c} \begin{array}{c} {TO}_{i\to *,t}(d)=\left(\frac{\sum_{i=1,j=1}^{n}{\tilde{\varphi }}_{ij,t}(d)}{\sum_{i=1,j=1}^{n}{\tilde{\varphi }}_{ij,t}(\infty )}\left(\sum_{j=1,i\neq j}^{n}{\tilde{\varphi }}_{ji,t}(d)\right)\right)\times 100 \end{array} \end{array}$$ (14)

$$\begin{array}{c} \begin{array}{c} {FROM}_{i\leftarrow *,t}(d)=\left(\frac{\sum_{i=1,j=1}^{n}{\tilde{\varphi }}_{ij,t}(d)}{\sum_{i=1,j=1}^{n}{\tilde{\varphi }}_{ij,t}(\infty )}\left(\sum_{j=1,i\neq j}^{n}{\tilde{\varphi }}_{ij,t}(d)\right)\right)\times 100 \end{array} \end{array}$$ (15)

$$\begin{array}{c} \begin{array}{c} {NET}_{i,t}(d)={TO}_{i\to *,t}(d)-{FROM}_{j\leftarrow *,t}(d) \end{array} \end{array}$$ (16)

$$\begin{array}{c} \begin{array}{c} {NPDC}_{ij,t}(d)=\left[\left(\frac{\sum_{i=1,j=1}^{n}{\tilde{\varphi }}_{ji,t}(d)}{\sum_{i=1,j=1}^{n}{\tilde{\varphi }}_{ij,t}(\infty )}{\tilde{\varphi }}_{ji,t}(d)\right)-\left(\frac{\sum_{i=1,j=1}^{n}{\tilde{\varphi }}_{ij,t}(d)}{\sum_{i=1,j=1}^{n}{\tilde{\varphi }}_{ij,t}(\infty )}{\tilde{\varphi }}_{ij,t}(d)\right)\right]\times 100 \end{array} \end{array}$$ (17)

Tr{⋅} denotes the trace operator.

### 3.2. Data

This study focuses on the Geopolitical Risk Index (GPR), crude oil markets, and chemical markets, and systematically analyzes the risk spillover effects among these markets. The GPR proposed by Caldara and Iacoviello [27] is employed as a proxy variable to quantify the time-varying nature and intensity of geopolitical risk. As an upstream commodity and a key raw material for many basic chemical products, crude oil price fluctuations directly affect the production costs of downstream chemical products. Drawing on the methodology of Hao et al. [47], this study selects three major crude oil markets: BRENT, INE, and WTI. Considering that China is the foremost producer and consumer of chemical products globally, the price volatility of its chemical commodities is not only significantly influenced by international crude oil prices but also exhibits high representativeness and policy sensitivity. Therefore, this study conducts an empirical analysis using the closing prices of chemical commodity futures traded on China’s futures markets, including bitumen (Bi), linear low-density polyethylene (LLDPE), polypropylene (PP), purified terephthalic acid (PTA), and polyvinyl chloride (PVC). Given that China’s INE market was officially launched in March 2018, the sample period spans from March 2018 to December 2024, with daily data used for empirical analysis. All raw data are collected from the Wind database, of which BRENT and WTI are global market data, and the rest are Chinese market data.

## 4. Empirical analysis

This part performs a series of empirical analyses based on the previously developed models and the organized dataset. This study employs a time-frequency domain approach based on the TVP-VAR model to construct a time-varying frequency-domain risk spillover network model. The model measures the risk linkages between GPR and crude oil together with its derivatives across short-term, medium-term, and long-term horizons. By calculating both static and dynamic spillover effects and constructing spillover network graphs, the study further illustrates the magnitude and transmission paths of risk within the network. In addition, the study applies an asymmetric TVP-VAR model to assess the risk linkages between GPR and crude oil together with its derivatives, and further explores the characteristics of spillover effects under conditions of risk asymmetry.

### 4.1. Descriptive statistics

Given the inconsistency in trading dates across markets, the sample data were initially filtered to ensure the continuity of each time series. Subsequently, the data were log-transformed, and the logarithmic returns were calculated accordingly. Table 1 presents the descriptive statistics for all variables. The results indicate that all variables exhibit typical time series characteristics, with means close to zero, but significant differences in volatility and distributional properties. The standard deviation of GPR is 0.4456, substantially higher than that of other commodities, and it exhibits a pronounced fat-tailed distribution, indicating a relatively higher level of risk. The oil markets (e.g., WTI and BRENT) show strong negative skewness (−2.92 and −2.07, respectively) and high kurtosis (67.34 and 30.79), suggesting that crude oil markets are more prone to frequent and sharp downturns. The chemical markets (e.g., LLDPE, PP, and PVC) exhibit relatively lower volatility, yet still show some degree of fat-tailed behavior. The ADF test results for all series are statistically significant, indicating stationarity and providing a sound basis for the subsequent spillover effect analysis.

**Table 1:** Descriptive statistics.

	GPR	BRENT	INE	WTI	Bi	LLDPE	PP	PTA	PVC
mean	−0.00015	0.00004	0.00014	0.00006	0.00019	−0.00005	−0.00013	−0.00009	−0.00018
std	0.4456	0.0276	0.0229	0.0354	0.0217	0.0139	0.0154	0.0160	0.0173
min	−3.00	−0.37	−0.14	−0.60	−0.15	−0.13	−0.21	−0.11	−0.17
max	2.34	0.19	0.13	0.32	0.14	0.12	0.10	0.08	0.12
kurtosis	2.23	30.79	4.89	67.34	8.78	17.69	39.15	5.53	17.40
skew	−0.15	−2.07	−0.16	−2.92	−0.24	−0.39	−2.67	−0.43	−0.50
JB	342.70***	65511.65***	1631.59***	310125.97***	5245.75***	21291.03***	105945.49***	2127.99***	20614.83***
ADF	−17.18***	−6.84***	−35.52***	−7.21***	−17.76***	−30.41***	−14.53***	−27.29***	−13.20***
N	1629	1629	1629	1629	1629	1629	1629	1629	1629

Note: ADF represents the Augmented Dickey-Fuller unit root test statistic; JB represents the Jarque-Bera test statistic;*, **, *** represent the significance at the 10%, 5%, and 1% levels.

### 4.2. Analysis of static risk spillover effects

To investigate the spillover effects between GPR, crude oil, and chemical markets, this study selects a lag order of 1 for the VAR model based on the Schwarz Information Criterion (SIC). Following the approach of Tiwari et al. [48], the forecast horizon for the variance decomposition is set at 100. The results of total spillover effects in the time domain across markets are reported in Table 2.

**Table 2:** Total static spillover effect in the time domain.

	GPR	BRENT	INE	WTI	Bi	LLDPE	PP	PTA	PVC	FROM
GPR	92.83	1.11	1.60	1.10	0.54	0.66	0.80	0.69	0.67	7.17
BRENT	0.35	49.09	2.30	42.74	1.15	1.14	0.62	1.68	0.94	50.91
INE	0.77	22.16	45.37	21.02	3.22	0.73	1.21	4.41	1.12	54.63
WTI	0.34	42.86	2.34	49.35	1.00	1.05	0.66	1.69	0.70	50.65
Bi	0.69	8.31	4.04	8.63	68.49	1.15	1.45	5.32	1.92	31.51
LLDPE	0.64	3.99	1.07	3.72	0.97	65.77	13.06	2.79	8.00	34.23
PP	0.63	3.31	1.30	3.49	1.54	12.61	66.33	4.30	6.50	33.67
PTA	0.44	12.09	4.02	11.42	4.32	2.74	3.15	59.83	1.98	40.17
PVC	0.61	2.29	0.58	1.91	1.23	8.90	7.49	2.65	74.33	25.67
TO	4.46	96.12	17.27	94.02	13.97	28.98	28.44	23.52	21.83	TCI
Net	−2.71	45.21	−37.37	43.37	−17.54	−5.25	−5.23	−16.64	−3.84	36.51

Note: The diagonal values in the table represent the current risk level of each risk variable in relation to its own lagged values.. The “TO” row indicates the extent to which a given risk variable contributes spillover effects to other variables. The “FROM” column reflects the extent to which a risk variable receives spillovers from other variables, and can be used to identify risk receivers and core transmission paths within the spillover network. The “Net” row represents the net spillover effect of the corresponding risk variable. TCI measures the overall magnitude and strength of spillovers within the entire system. Same applies to subsequent tables. The original data is sourced from Wind.

As shown in Table 2, the total spillover effect among markets is 36.51%, indicating a significant risk contagion between GPR, crude oil, and chemical markets. This result may be jointly driven by the intensification of geopolitical risks and the increasing financialization of the crude oil market. Among the spillover effects from GPR to the oil and chemical markets, the INE market exhibits the highest intensity, suggesting that it is more sensitive to geopolitical risks. In comparison, the international crude oil markets are particularly responsive to geopolitical risks. This phenomenon is primarily due to the fact that crude oil, as a globally traded asset with high liquidity and large trading volume, tends to experience sharp volatility under extreme market sentiments such as wars or energy crises. Such volatility is often interpreted by the media, policymakers, and market analysts as a signal of heightened geopolitical uncertainty.

The net spillover results show that the BRENT and WTI markets have net spillover effects of 45.21% and 43.37%, respectively, indicating that crude oil markets act as primary risk transmitters in the cross-market spillover structure. The net spillover effects of GPR and the chemical markets are negative, suggesting that they mainly serve as risk receivers within the system-highly sensitive to external shocks yet lacking the capacity to transmit risk to other parts of the system. This phenomenon reveals the passive nature of both GPR and the chemical markets and further reflects their heavy reliance on upstream markets such as crude oil.

To examine the spillover effects between GPR, crude oil, and chemical markets across various frequency domains, this study applies the TVP-VAR-BK spillover framework to compute the spillover effects under short-, medium-, and long-term conditions. Following Deng and Xu [49], the sample frequency domain is divided into high, medium, and low frequency bands, corresponding to short-, medium-, and long-term horizons, respectively. The spillover results across different frequency domains are reported in Table 3.

**Table 3:** Total static spillover effect in the frequency domain.

	GPR	BRENT	INE	WTI	Bi	LLDPE	PP	PTA	PVC	FROM
Short-term										
GPR	85.86	1.04	1.51	1.02	0.48	0.61	0.71	0.62	0.62	6.62
BRENT	0.3	39.72	1.77	34.49	0.91	0.94	0.51	1.27	0.75	40.94
INE	0.6	15.31	34.13	14.39	2.11	0.52	0.83	2.69	0.9	37.35
WTI	0.3	35.15	1.9	40.62	0.81	0.91	0.52	1.3	0.56	41.46
Bi	0.58	5.94	2.87	6.27	54.65	0.85	1.1	3.76	1.49	22.86
LLDPE	0.56	2.68	0.79	2.5	0.73	53.87	9.84	1.88	6.39	25.38
PP	0.52	2.2	0.89	2.33	1.15	9.68	52.52	2.97	4.95	24.7
PTA	0.35	8.29	2.9	7.82	3.24	1.95	2.36	42.98	1.41	28.32
PVC	0.55	1.78	0.46	1.48	0.93	6.95	5.97	1.98	60.89	20.11
TO	3.76	72.39	13.1	70.3	10.38	22.42	21.85	16.48	17.07	TCI
Net	−2.86	31.45	−24.25	28.84	−12.49	−2.95	−2.86	−11.84	−3.05	27.53
Medium-term										
GPR	5.19	0.05	0.06	0.06	0.04	0.04	0.06	0.05	0.04	0.41
BRENT	0.03	6.89	0.39	6.07	0.17	0.14	0.09	0.3	0.14	7.32
INE	0.12	4.99	8.23	4.83	0.8	0.15	0.27	1.25	0.16	12.58
WTI	0.03	5.68	0.32	6.44	0.14	0.11	0.1	0.29	0.1	6.77
Bi	0.08	1.73	0.86	1.72	10.17	0.22	0.26	1.14	0.31	6.32
LLDPE	0.06	0.96	0.21	0.89	0.17	8.75	2.35	0.65	1.18	6.46
PP	0.09	0.8	0.3	0.83	0.28	2.13	10.1	0.96	1.13	6.52
PTA	0.07	2.77	0.82	2.62	0.79	0.57	0.57	12.24	0.42	8.63
PVC	0.04	0.37	0.09	0.31	0.22	1.43	1.12	0.49	9.9	4.07
TO	0.52	17.36	3.04	17.34	2.62	4.79	4.82	5.12	3.48	TCI
Net	0.11	10.03	−9.54	10.56	−3.7	−1.66	−1.71	−3.51	−0.59	6.56
Long-term										
GPR	1.79	0.02	0.02	0.02	0.01	0.01	0.02	0.02	0.01	0.14
BRENT	0.01	2.48	0.14	2.18	0.06	0.05	0.03	0.11	0.05	2.64
INE	0.05	1.86	3.01	1.8	0.3	0.06	0.1	0.47	0.06	4.7
WTI	0.01	2.02	0.12	2.29	0.05	0.04	0.04	0.1	0.04	2.42
Bi	0.03	0.64	0.32	0.64	3.67	0.08	0.09	0.42	0.11	2.33
LLDPE	0.02	0.36	0.08	0.33	0.06	3.15	0.87	0.25	0.43	2.4
PP	0.03	0.3	0.11	0.32	0.11	0.79	3.71	0.36	0.42	2.45
PTA	0.02	1.04	0.31	0.98	0.29	0.21	0.21	4.61	0.16	3.22
PVC	0.01	0.13	0.03	0.11	0.08	0.52	0.41	0.18	3.55	1.48
TO	0.19	6.37	1.12	6.38	0.97	1.76	1.78	1.92	1.28	TCI
Net	0.05	3.73	−3.58	3.96	−1.36	−0.64	−0.67	−1.29	−0.2	2.42

As shown in Table 3, the spillover effects among GPR, crude oil, and chemical markets are 27.53%, 6.56%, and 2.42% in the short-, medium-, and long-term frequency bands, respectively. The sum of these frequency-domain spillovers equals the total spillover observed in the time domain. Spillover effects among markets are substantially higher in the high-frequency band compared to the medium- and low-frequency bands. This suggests that the spillovers between GPR and the crude oil and chemical markets are primarily driven by high-frequency components, with most spillover effects occurring in the short term. This pattern can be attributed to the rapid dissemination of information related to geopolitical risk events, the strong and immediate market reactions they trigger, and the prompt pricing mechanisms of asset markets in response to unexpected events.

In summary, there exists significant risk contagion between GPR, crude oil, and chemical markets. BRENT and WTI serve as major risk transmitters, while the chemical markets and GPR primarily act as risk receivers, highlighting their dependence on upstream markets and their passive roles within the system. In the frequency-domain analysis, short-term spillover effects are markedly stronger than those in the medium- and long-term, suggesting that the system’s risk transmission is predominantly driven by high-frequency factors. This reflects the rapid dissemination of information during geopolitical events and the markets’ immediate response to such developments.

### 4.3. Analysis of dynamic risk spillover effects

Although static risk spillover effects can depict the overall characteristics of the GPR, bulk energy, and chemical commodity markets, they fail to capture the time-varying features of these markets. To gain a more profound comprehension of the risk spillover effects between markets, this paper provides a detailed analysis of their time-varying characteristics across both time and frequency domains.

**Fig 1:**
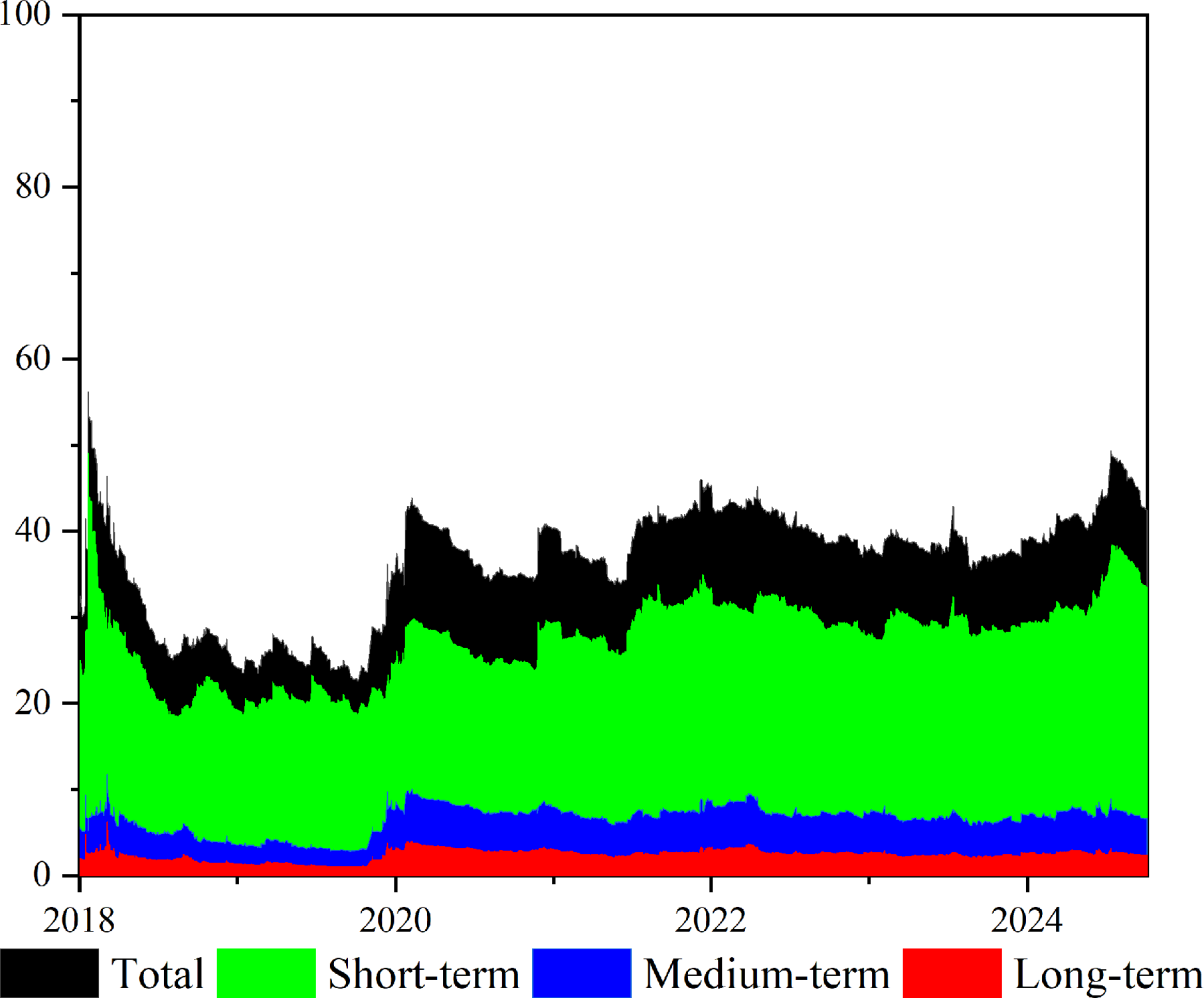
Dynamic total spillover effect in time-frequency domain. Total spillover effect (black shaded area); short-term frequency spillover effect (green shaded area); medium-term frequency spillover effect (blue shaded area); and long-term frequency spillover effect (red shaded area). The original data is sourced from Wind.

As shown in Fig 1, the total spillover effects between GPR, crude oil, and chemical markets exhibit significant time-varying characteristics, fluctuating between 20% and 60% in the time domain and being highly susceptible to major geopolitical events and policy shocks. For example, during the U.S.-China trade tensions in 2018, the risk spillover between GPR, the crude oil and chemical markets rose markedly. Specifically, the tariff dispute in April 2018 triggered a spillover peak of over 56%; following the Russia-Ukraine conflict in March 2022, the spillover level climbed again to 46.0%. Although spillover peaks remain closely tied to major events, markets have gradually improved their capacity to address extreme risks, resulting in an overall decline in volatility, which indicates high sensitivity in the early stages and stronger adaptability in the later stages.

From a frequency-domain perspective, the total spillover effects in the time domain between GPR, crude oil, and chemical markets closely mirror those observed in the short-term frequency domain. In addition, spillover effects are substantially higher in the short-term frequency domain compared to the medium- and long-term domains. This result further suggests that, during most periods, the spillover effects across markets are predominantly driven by high-frequency factors, and the risk linkages between GPR, the crude oil and chemical markets are more strongly influenced by short-term shocks. As the time horizon extends, the spillover effects gradually diminish and tend to stabilize. The heterogeneity of spillover effects across frequency domains highlights the high degree of financialization and trading activity in the crude oil and chemical markets, which significantly amplifies risk transmission in the short term. In contrast to the gradual developments over the medium and long term, shocks triggered by geopolitical events tend to be rapidly and intensively released in the short term, thus shaping a spillover pattern dominated by high-frequency drivers.

**Fig 2:**
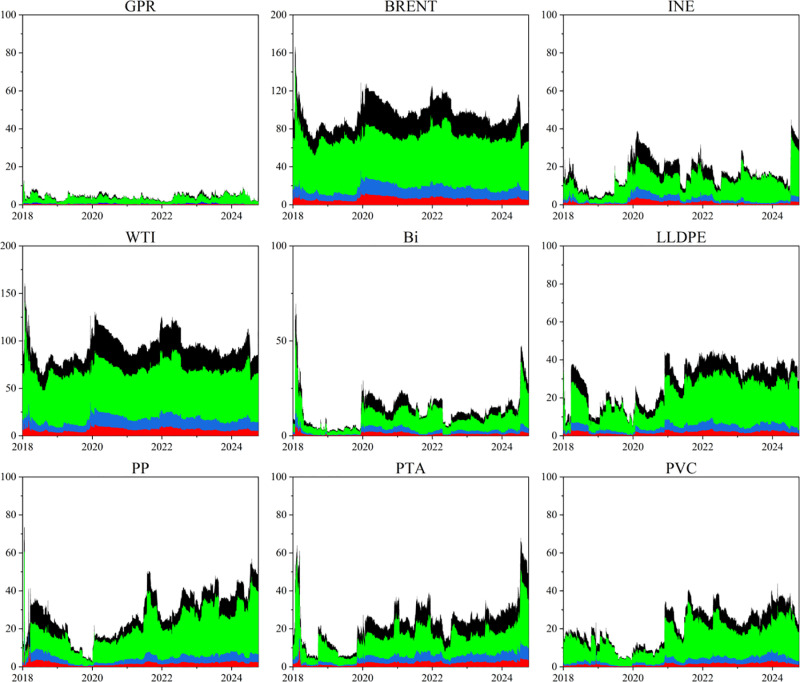
Dynamic spillover effect in time-frequency domain. The original data is sourced from Wind.

**Fig 3:**
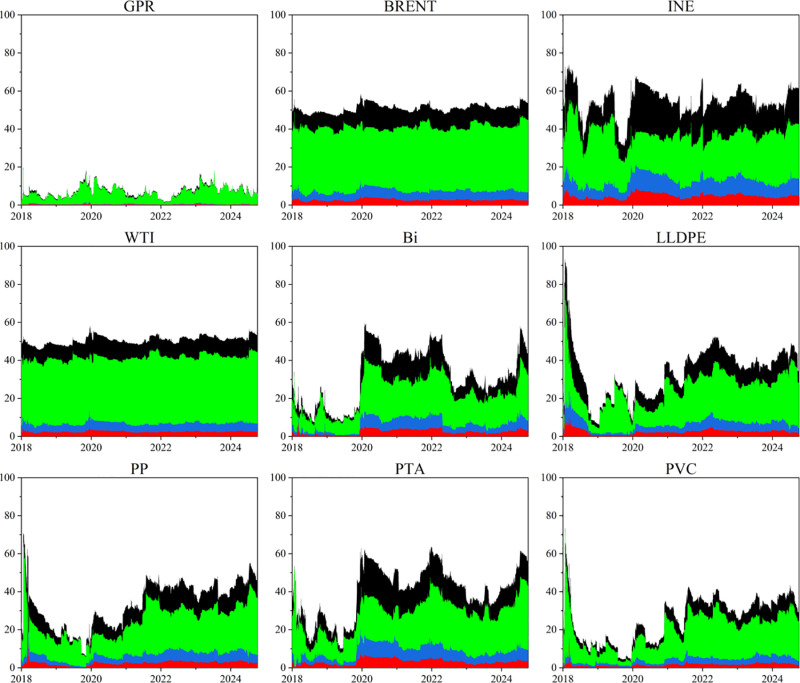
Dynamic spill-in effect in time-frequency domain. The original data is sourced from Wind.

This paper plots the directional spillover trends for each market across both time and frequency domains to investigate the time-varying characteristics of spillover effects among individual markets within the GPR, bulk energy, and chemical commodity markets system. Analyzing the dynamic fluctuations in risk spillover effects allows for the effective identification of primary sources of risk spillover throughout various periods, while examining risk spill-in effects largely reflects each market’s ability to withstand risk shocks. The results are shown in Figs 2 and 3.

From the perspective of risk spillovers (TO), BRENT and WTI significantly outperform other markets, jointly contributing the majority of cross-market risk transmission over the entire sample period. The strong spillover capacity of BRENT and WTI underscores their central roles in risk transmission: on one hand, as global pricing benchmarks for crude oil, their prices are highly sensitive to OPEC+ production decisions and geopolitical conflicts (such as the recent Russia-Ukraine war), and respond swiftly. On the other hand, their price fluctuations can be rapidly transmitted to downstream petrochemical markets via crude oil import structures and international trade flows. In the downstream markets, LLDPE and PP exhibit a certain “relay” function —— once crude oil price shocks reach the petrochemical segment, these intermediate products further propagate the risk to adjacent products and downstream industrial chains. In contrast, products such as PTA, PVC, and Bi contribute relatively less to overall risk transmission. Notably, the risk spillover level of the INE market is significantly lower than that of BRENT and WTI, indicating its role as a “risk receiver with passive pricing” rather than an “active transmitter”. The direct spillover effect of the GPR on other markets is relatively weak, suggesting that its transmission mechanism primarily operates by first influencing crude oil prices, which then cascade downstream through the oil market.

From the perspective of risk spill-in (FROM), domestic markets and downstream products are more likely to act as “absorbers” of risk. The average risk reception level of the INE market exceeds that of BRENT and WTI. On the one hand, although the INE’s pricing mechanism has become increasingly mature, it remains highly sensitive to price fluctuations in BRENT and WTI. On the other hand, given the high external dependence of China’s crude oil market, it is difficult for the INE to absorb price shocks caused by international political and economic events independently. Among downstream petrochemical products, the risk reception capacity decreases in the following order: PTA, LLDPE, PP, Bi, and PVC. This suggests that, under conditions of sharp volatility in international oil prices caused by unexpected events, downstream chemical enterprises face dual pressures of soaring raw material costs and revaluation of corporate worth. Meanwhile, the crude oil market shall handle exogenous shocks from geopolitical events while also absorbing secondary fluctuations driven by downstream demand feedback, resulting in a compound pattern of risk transmission.

**Fig 4:**
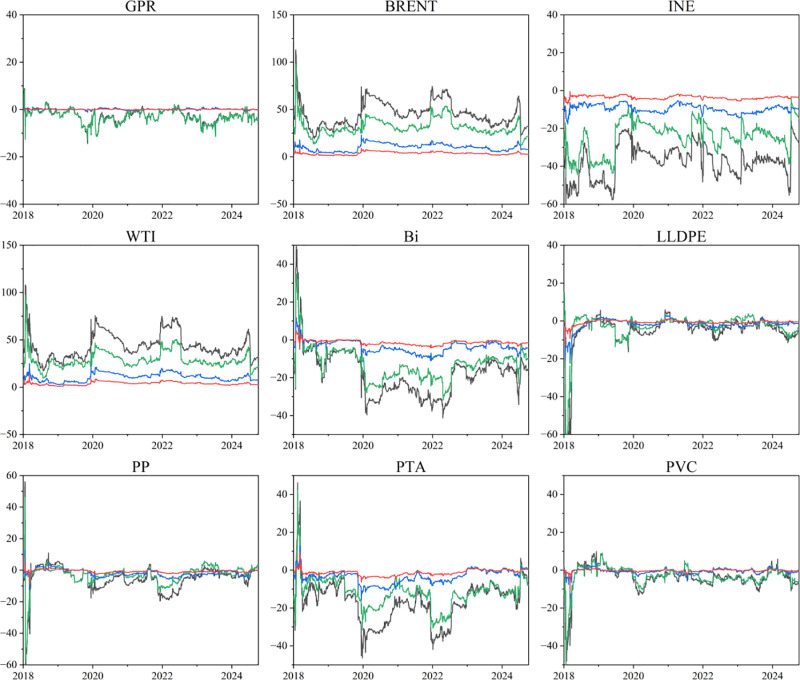
Dynamic net spillover effect in time-frequency domain. The original data is sourced from Wind.

To examine the time-varying roles and positions of individual markets in the risk spillovers between GPR, crude oil, and chemical markets, this study plots the dynamic trends of net spillover effects in both time and frequency domains, as presented in Fig 4. From the perspective of the time domain and the high-frequency domain, the net spillover effects of each market exhibit broadly consistent trends with substantial volatility. BRENT and WTI in the international oil market act as key risk transmitters, while INE, the GPR, and downstream chemical markets primarily serve as net recipients. Frequency-domain analysis further shows that downstream chemical products exhibit the highest level of risk reception in the short term, while their reception capacity declines significantly over the medium and long term. The net spillover index of GPR turns slightly positive in the medium- and long-term stages, indicating that political risk is typically transmitted only after the initial shock has been partially absorbed by the oil market and chemical supply chain.

These characteristics are consistent with the INE’s initial illiquidity, the occurrence of negative oil prices in the WTI market in 2020, and the sharp oil price volatility triggered by the Russia-Ukraine conflict in 2022. These events highlight the significant short-term impact of oil prices on downstream markets and risk factors, as well as the tendency for medium- and long-term stabilization driven by shifts in supply-demand fundamentals and policy adjustments.

In summary, the risk spillover effects between GPR, crude oil, and chemical markets exhibit significant time variability and frequency-domain heterogeneity. Thus, H1 is proved. The level of spillovers increases markedly during geopolitical conflicts and major policy events, displaying sharp short-term volatility and a gradual return to stability over the long term. Risk transmission within this system reveals a hierarchical structure characterized by “upstream proactivity and downstream passivity”, shaped by the dynamic evolution of liquidity conditions, policy expectations, and market structure.

### 4.4. Analysis of the asymmetric risk spillover effects

Existing studies have shown that the network linkages between GPR and the crude oil and chemical markets exhibit asymmetry [40]. This study tests the asymmetric effects of risk spillovers between GPR and the crude oil and chemical markets by decomposing each risk variable into daily positive and negative risk components, specifically as follows:


$$\begin{array}{c} \begin{array}{c} S_{t}=\left\{ \;\;\;\begin{array}{c} 0,if\;y_{t}<0\\ 1,if\;y_{t}\geq 0 \end{array}\right.\; \end{array} \end{array}$$ (18)

$$\begin{array}{c} \begin{array}{c} y_{t}^{+}=S_{t}\cdot y_{t} \end{array} \end{array}$$ (19)

$$\begin{array}{c} \begin{array}{c} y_{t}^{-}=\left(1-S_{t}\right)\cdot y_{t} \end{array} \end{array}$$ (20)

where $y_{t}^{+}$ and $y_{t}^{-}$ represent the positive and negative daily degree of risk changes, respectively.

First, this study examines the static risk spillover effects between GPR, crude oil and chemical markets under risk asymmetry. Table 4 presents the risk spillover networks considering both positive and negative risk shocks. The total spillover index based on negative risk shocks is 37.84, which is higher than the 34.21 observed under positive shocks. This finding indicates that the risk spillover effects between GPR, the crude oil and chemical markets are asymmetric.

**Table 4:** Static risk spillover effects on considering risk asymmetries.

–	GPR	BRENT	INE	WTI	Bi	LLDPE	PP	PTA	PVC	FROM.
GPR	91.96	1.26	1.25	1.17	0.64	1.30	0.89	0.80	0.72	8.04
BRENT	0.42	47.83	2.64	41.20	1.88	1.42	1.01	2.33	1.28	52.17
INE	0.71	21.80	45.07	22.05	3.64	1.35	1.21	3.38	0.78	54.93
WTI	0.41	41.44	2.45	48.63	1.86	1.38	1.11	2.05	0.68	51.37
Bi	0.47	8.86	3.61	9.52	63.62	2.73	2.75	5.52	2.91	36.38
LLDPE	0.80	2.87	1.71	3.00	1.42	71.23	6.64	3.18	9.16	28.77
PP	0.78	3.01	1.34	3.19	2.49	10.13	66.42	6.03	6.62	33.58
PTA	0.70	11.82	2.62	11.14	4.55	4.57	4.39	56.83	3.37	43.17
PVC	0.48	1.66	0.91	1.40	1.78	16.53	6.01	3.39	67.83	32.17
TO	4.78	92.73	16.53	92.67	18.26	39.41	24.02	26.68	25.51	TCI
Net	−3.26	40.56	−38.40	41.30	−18.12	10.63	−9.57	−16.48	−6.66	37.84
+	GPR	BRENT	INE	WTI	Bi	LLDPE	PP	PTA	PVC	FROM
GPR	91.59	1.09	0.94	0.96	1.06	1.41	1.07	1.03	0.86	8.41
BRENT	0.36	50.51	2.02	41.78	1.05	0.81	0.82	1.83	0.81	49.49
INE	0.68	18.05	52.77	17.56	2.9	1.32	1.66	4.47	0.59	47.23
WTI	0.39	41.68	1.8	50.94	0.96	0.9	0.71	1.96	0.65	49.06
Bi	0.84	5.85	3.56	6.24	72.09	1.82	1.78	6.06	1.77	27.91
LLDPE	0.43	2.91	1.53	2.66	1.75	64.18	12.62	2.99	10.92	35.82
PP	0.49	2.59	1.2	3.03	1.83	11.75	70.39	3.15	5.57	29.61
PTA	0.57	8.04	4.08	8.61	4.88	2.85	2.47	66.46	2.04	33.54
PVC	0.5	1.85	0.51	1.74	0.93	12.42	6.49	2.4	73.18	26.82
TO	4.25	82.05	15.64	82.58	15.36	33.28	27.61	23.89	23.22	TCI
Net	−4.16	32.56	−31.59	33.52	−12.55	−2.54	−2	−9.64	−3.6	34.21

Under positive risk shocks, the BRENT and WTI markets dominate the spillover network. In the network experiencing negative risk shocks, the LLDPE market shifts from a net receiver to a net transmitter. This highlights the significantly increased importance of the LLDPE market in the asymmetric spillover network. This is because LLDPE is widely used in industries including packaging films and agricultural films, and its demand is highly sensitive to macroeconomic conditions and market expectations. In times of adverse economic forecasts or policy tightening, declining end-consumer expectations are rapidly reflected.

**Fig 5:**
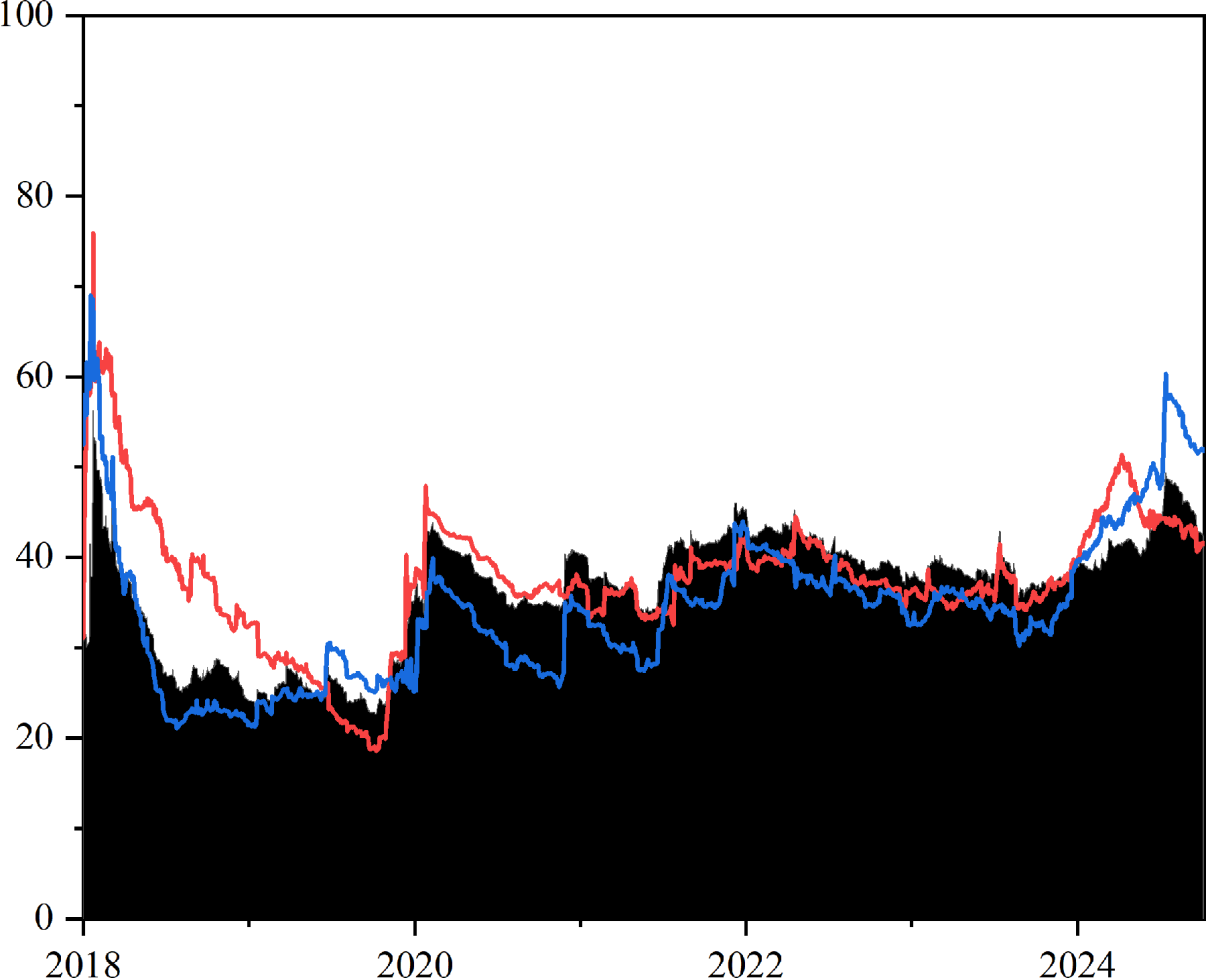
Asymmetric total risk spillover in the time domain. The original data is sourced from Wind.

This study also analyzes the dynamic evolution of the total risk spillover index between GPR, the crude oil and chemical markets under risk asymmetry. As shown in Fig 5, negative spillovers triggered by downside risk events exhibit significantly greater magnitudes and higher peaks compared to positive spillovers. For example, in April 2020, when WTI futures prices plunged to -$40, the negative spillover index spiked sharply —— far exceeding the corresponding positive spillovers, highlighting the intense downward contagion effect induced by the supply glut shock.

Subsequently, driven by expectations of OPEC+ production cuts, BRENT crude prices gradually recovered, with the corresponding positive spillover rising from 20 to 40, while negative spillovers declined to below 30. This indicates that while optimistic expectations generated sustained positive transmission effects, their intensity was markedly lower than that of negative shocks during crisis periods. In 2022, the Russia-Ukraine conflict caused an increase in BRENT oil prices, with negative spillovers dominating once more. Despite positive spillovers increased to some extent, their overall magnitude remained limited. Negative spillovers respond more rapidly and peak at higher levels, while positive spillovers exhibit notable lags and relatively milder transmission characteristics. To better illustrate these differences, Fig 5 further presents the net difference in total spillover indices by decomposing positive and negative risk conditions.

**Fig 6:**
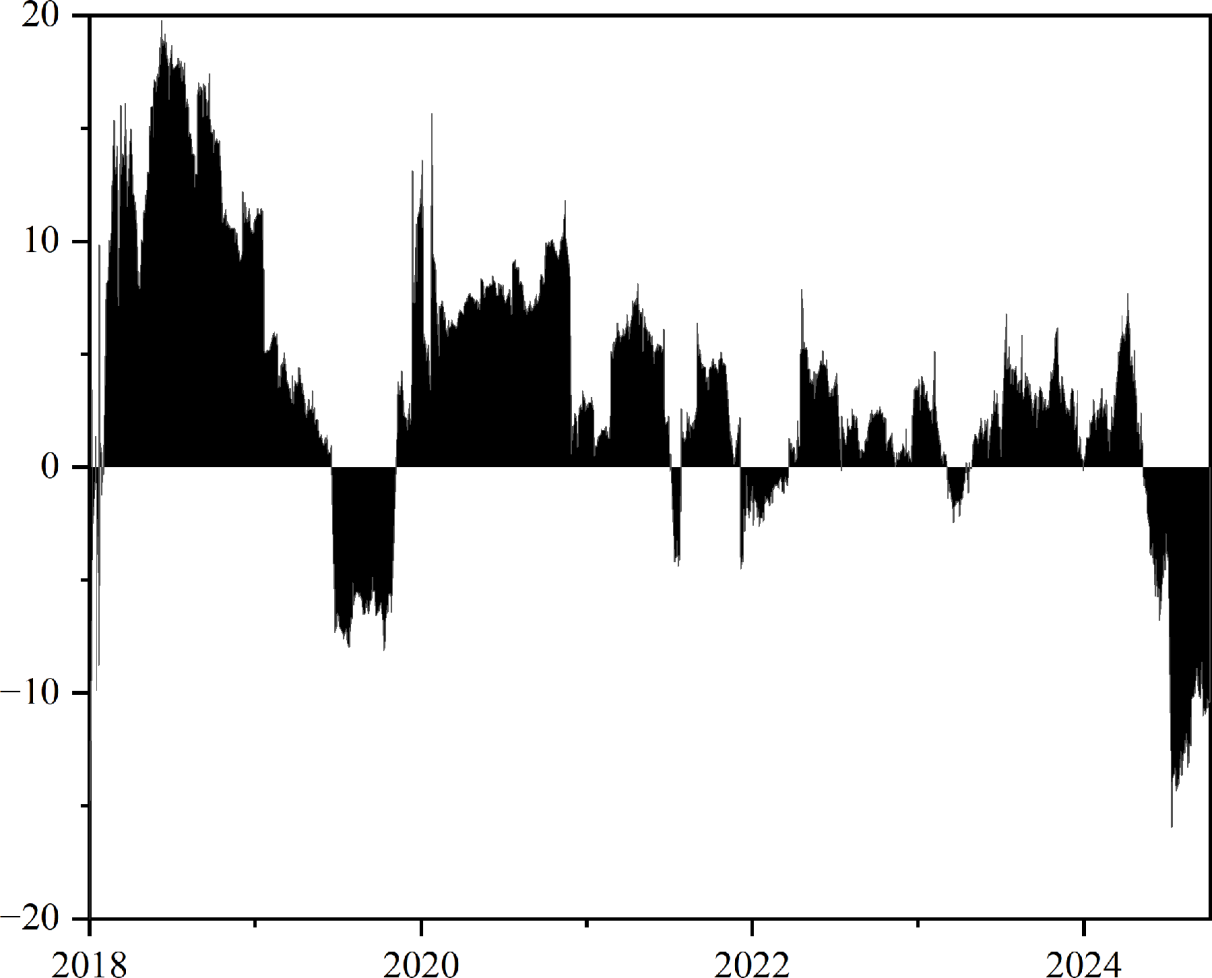
Asymmetric effects of total spillover considering risk asymmetry. The original data is sourced from Wind.

Fig 6 illustrates that over the majority of the sample period, the risk spillover network between GPR, crude oil, and chemical markets is predominantly driven by the network based on negative risk shocks. This phenomenon primarily arises because, in most cases, financial market participants, particularly risk-averse investors, tend to focus more on losses than on gains and exhibit exaggerated responses to negative risk shocks [50], leading the entire risk spillover network to respond more strongly to negative risks than to positive ones. However, in 2019 and 2024, the risk spillover network was instead dominated by the components based on positive risk changes. This phenomenon may be attributed to the persistently low levels of the GPR index over the past two years, resulting in a spillover network primarily shaped by positive risk dynamics.

In summary, the risk spillover effects between GPR, crude oil, and chemical markets exhibit significant asymmetry, with spillovers triggered by negative risk shocks generally stronger than those from positive shocks. Therefore, H2 is proved. Under negative event-driven conditions, such as the occurrence of negative WTI oil prices and the Russia-Ukraine conflict, the total system spillover index rises rapidly, with more intense transmission effects and higher peaks. In contrast, positive risk spillovers are relatively moderate and exhibit delayed transmission.

### 4.5. Risk spillover network analysis

**Fig 7:**
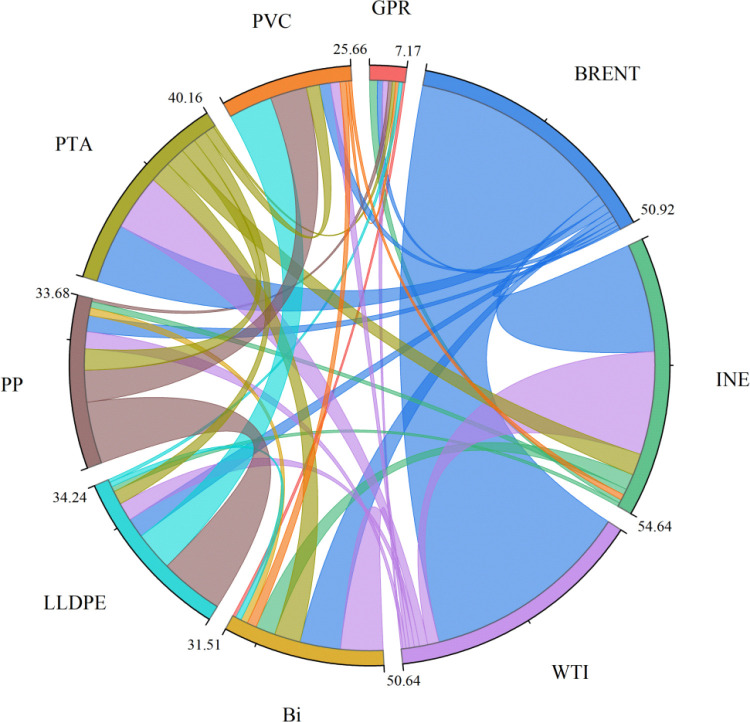
Network structure without considering risk asymmetry. The original data is sourced from Wind.

To further investigate the network interconnections among the GPR, crude oil, and chemical markets, this paper establishes a spillover network based on net pairwise spillover values to depict the cross-market risk transmission paths, as shown in Fig 7. The size of each node reflects the magnitude of the market’s net pairwise spillover value, while the size and direction of the edges represent the intensity and direction of spillovers between market pairs. Analyzing the relationship between GPR and crude oil, and chemical markets reveals significant differences in the strength of their interconnections. This indicates a clear asymmetry in the transmission of information and risk influence within the spillover network formed by GPR, crude oil, and chemical markets, with certain markets playing dominant roles in the system.

**Fig 8:**
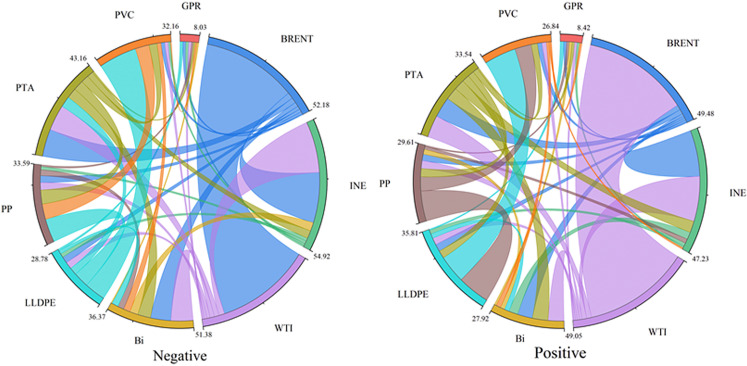
Network structure considering risk asymmetry. The original data is sourced from Wind.

Specifically, as shown in Fig 8, when risk asymmetry is not considered, the connections between BRENT, WTI, and PTA and other markets are visualized using distinct colors, reflecting their role in transmitting risk to other markets. On one hand, the rapid advancement of 5G technology and electronics has significantly increased demand for electronic components, thereby driving a surge in market demand for key raw materials like PTA. This shift in demand has led to pronounced fluctuations in PTA prices, which have further spilled over into other markets. On the other hand, in the context of GPR fluctuations, BRENT and WTI-as global benchmarks for oil pricing exert direct influence on the risk levels of global oil and chemical markets. Risk is primarily transmitted from the BRENT, INE, and PTA markets to other chemical commodity markets.

Fig 8 illustrates that, under the condition of risk asymmetry, the connections between BRENT, INE, and WTI crude oil futures markets and other markets are visualized using different colors, indicating that crude oil futures markets continue to play a central role as risk transmitters within the system, while chemical commodity markets more frequently function as risk receivers. This further demonstrates that, under extreme shock conditions, price volatility in oil markets significantly affects chemical products that strongly rely on crude oil for production, with risk predominantly transmitted from the oil to the chemical markets. When accounting for risk asymmetry, the LLDPE market, previously a risk receiver, begins to transmit more risk to other chemical markets, gradually evolving into a risk contributor. This phenomenon highlights the complex and dynamic interactions among GPR, energy, and chemical commodity markets in a highly interconnected global economy. The functional roles of individual markets may shift in response to external shocks.

In Summary, the risk spillover effects between GPR, the crude oil and chemical markets exhibit clear asymmetry under positive and negative shocks. The total spillover index is significantly higher under negative risk shocks than under positive ones, indicating that the system reacts more intensely to downside risks, with faster transmission and higher peak values. In the negative spillover network, the LLDPE market shifts from being a passive recipient to an active transmitter, reflecting its high sensitivity to changes in macroeconomic expectations. Overall, the market’s overreaction mechanism in response to negative shocks makes negative risk the primary driving force in the spillover network, further revealing the prevalent risk aversion behavior and asymmetric response patterns in financial markets. Therefore, H3 is proved.

### 4.6. Discussion

The findings of this study enrich and extend previous research on GPR and commodity markets. Similar to the findings of Coskun et al. [11], they confirm the significant role of volatility in driving risk spillover effects, and are consistent with the theoretical premise of Caldara and Iacoviello [27] that geopolitical uncertainty has a significant impact on macroeconomic dynamics. This study distinguishes itself from previous studies, which primarily focus on the relationship between static variables [25,26], by revealing the dynamic evolution characteristics of time-frequency spillovers and highlighting the time-varying nature of risk transmission across different market conditions. Then, although the studies of Qin et al. [40] and Cheng et al. [18] have explored the asymmetric effects of GPR, this study further demonstrates that the structural path of risk contagion changes when considering risk asymmetry. GPR becomes an important catalyst for risk contagion. These findings further expand the research of relevant scholars [2] by providing empirical evidence for the network structure of market risk transmission from the perspective of the industrial chain, providing a more comprehensive perspective for understanding the complex mechanism of GPR affecting the energy and chemical market, and effectively implementing risk management strategies.

## 5. Conclusion and policy implications

### 5.1. Conclusion

Based on the empirical findings, this section summarizes the analytical process and key results:

(1) There are significant risk spillover effects between GPR, crude oil, and chemical markets. Empirical results from the VAR and TVP-VAR-BK models reveal strong systemic linkages among the three, with BRENT and WTI acting as major risk transmitters, while GPR and chemical markets serve primarily as risk receivers —— highlighting an “upstream-active to downstream-passive” risk transmission structure.
(2) The spillover effects exhibit significant time variation and frequency-domain heterogeneity. Short-term shocks caused by geopolitical conflicts and major policy events significantly strengthen inter-market risk connections. Frequency-domain analysis shows that high-frequency spillovers dominate risk transmission, reflecting the market’s immediate pricing response to sudden information.
(3) The risk spillovers are markedly asymmetric. Negative shocks such as the plunge in WTI prices into negative territory and the Russia-Ukraine war, generate stronger spillover effects than positive shocks, with higher peaks and stronger transmission capacity. This reveals the market’s heightened sensitivity and asymmetric responses under adverse conditions.
(4) The importance of LLDPE rises significantly within the negative risk spillover network, indicating its high sensitivity to changes in macroeconomic expectations. Under negative economic expectations, LLDPE shifts from a risk receiver to a risk transmitter, acting as a “relay” of risk within the industrial chain. This shift further highlights the vulnerability and feedback mechanisms of the chemical market during periods of extreme events.

The limitations of this study are as follows: First, the selection of the bulk energy market did not consider other energy markets such as natural gas and coal. Future research could benefit from incorporating natural gas and coal markets to provide a more comprehensive analysis of tail risk contagion. Second, the TVP-VAR model may be subject to the risk of high complexity and overfitting. Future research could employ dimension reduction techniques based on Elastic Net to estimate, enabling time-varying estimation for high-dimensional data.

### 5.2. Policy implications

Based on the above conclusions, this paper puts forward the following policy implications. First, geopolitical risks and volatility contagion between bulk energy and chemical commodity markets exhibit significant time-varying asymmetry, lag, and cyclicality, with short-term risk spillovers as the dominant driver. Therefore, policies should prioritize expectation management and dynamic regulation. On the one hand, multi-dimensional investor sentiment monitoring platforms should be built to strengthen the high-frequency tracking of short-term market sentiment fluctuations, and regular industry reports should be released to mitigate the cumulative effect of short-term risk spillovers. On the other hand, a differentiated risk response mechanism should be established, setting quantitative trigger thresholds for negative shocks, while conducting dynamic assessments for positive shocks to avoid excessive policy intervention. Meanwhile, the asymmetry of risk spillovers is predominantly negative in most cases, indicating that the entire risk network is more vulnerable to downward risks. It is necessary to focus on the downward trend of the market and strengthen early warning and prevention of short-term extreme events.

Second, within the risk spillover network, the bulk energy market acts as a risk transmitter, while the chemical market serves as a risk receiver. A dual strategy of “source prevention and control-terminal strengthening” should be implemented. In international bulk energy markets, cross-border collaborative regulation of BRENT and WTI crude oil markets should be strengthened, and joint international crude oil price monitoring mechanisms should be promoted. Simultaneously, domestic crude oil futures hedging tools should be enriched to guide enterprises to hedge price risks. For domestic chemical markets, focusing on key varieties such as LLDPE, full-industrial-chain digital monitoring platforms should be established to achieve dynamic early warning of supply and demand risks. In addition, a hierarchical response system of “national strategic reserves - enterprise commercial reserves” should be established to quickly adjust supply and demand during market fluctuations. Emergency logistics network within chemical parks should be developed to enhance supply chain resilience.

Third, as risks propagate along the “bulk energy - chemical commodities” pathway, stronger coordination is needed among monitoring, investment and imports. The quantitative index system for geopolitical risks should be improved, integrating geopolitical conflict intensity, policy uncertainty indices and energy chemical market indicators into the macro-prudential assessment framework to achieve dynamic risk tracking. At the same time, investor education should be enhanced to guide cross-asset portfolio allocation to fully consider the impact of geopolitical conflicts on energy and chemical sectors. Diversified import channels should also be expanded for varieties such as asphalt and polypropylene. In addition, a rapid response mechanism for geopolitical conflicts should be established, allowing for dynamic adjustment of tariffs and quotas according to the scale of conflicts, thereby mitigating the adverse impact of geopolitical risks on commodity markets.

## Supporting information

S1 Data Code and data. (ZIP)
