# Correction: Does geopolitical risks drive the extreme spillovers of bulk energy and chemical commodity

**DOI:** 10.1371/journal.pone.0335169

**Published:** 2025-10-22

**Authors:** Zhang Tao, Tianzhuang Li, Yadi Chen, Xiaoyue Huang

The authors, Zhang Tao, Tianzhuang Li, Yadi Chen and Xiaoyue Huang should not have been attributed equal contribution to this work.
